# Intraluminal Flagellin Differentially Contributes to Gut Dysbiosis and Systemic Inflammation following Burn Injury

**DOI:** 10.1371/journal.pone.0166770

**Published:** 2016-12-01

**Authors:** Logan Grimes, Allie Doyle, Aaron L. Miller, Richard B. Pyles, Gabor Olah, Csaba Szabo, Sarah Hoskins, Tonyia Eaves-Pyles

**Affiliations:** 1 Departments of Microbiology and Immunology, University of Texas Medical Branch, Galveston, Texas, United States of America; 2 Pediatrics, University of Texas Medical Branch, Galveston, Texas, United States of America; 3 Anesthesiology, University of Texas Medical Branch, Galveston, Texas, United States of America; 4 Shriners Hospitals for Children, Galveston, Texas, United States of America; Universidade Federal de Sao Paulo, BRAZIL

## Abstract

Burn injury is associated with a loss of gut barrier function, resulting in systemic dissemination of gut-derived bacteria and their products. The bacterial protein and TLR5 agonist, flagellin, induces non-specific innate immune responses. Because we detected flagellin in the serum of burn patients, we investigated whether gut-derived flagellin was a primary or secondary contributor to intestinal dysfunction and systemic inflammation following burn injury. The apical surface of polarized human intestinal epithelial cells (IECs), Caco-2BBe, were exposed to 50 or 500 ng of purified flagellin and 1 x 10^5^ of an intestinal *E*. *coli* (EC) isolate as follows: 1) flagellin added 30 min prior to EC, 2) flagellin and EC added simultaneously, or 3) EC added 30 min prior to flagellin. Our results showed that luminal flagellin and EC modulated each other's biological actions, which influenced their ability to induce basolateral secretion of inflammatory cytokines and subsequent translocation of bacteria and their products. A low dose of flagellin accompanied by an enteric EC in the lumen, tempered inflammation in a dose- and time-dependent manner. However, higher doses of flagellin acted synergistically with EC to induce both intestinal and systemic inflammation that compromised barrier integrity, increasing systemic inflammation following burn injury, a process we have termed flagellemia. In a murine model of burn injury we found that oral gavage of flagellin (1 μg/mouse) significantly affected the gut microbiome after burn injury. In these mice, flagellin disseminated out of the intestine into the serum and to distal organs (mesenteric lymph nodes and lungs) where it induced secretion of monocyte chemoattractant protein (MCP-1) and CXCL1/KC (mouse equivalent of human IL-8) at 24 and 48h post-burn. Our results illustrated that gut-derived flagellin alone or accompanied by a non-pathogenic enteric EC strain can function as an initiator of luminal and systemic inflammation following burn injury.

## Introduction

Following burn injury, intestinal barrier failure allows luminal bacteria and their products to escape the confines of the gut to the mesenteric lymph nodes (MLN). Following arrival in the MLN these molecules then disseminate systemically [1–3]. Enteric bacteria and their products, including bacterial lipopolysaccharide (LPS), have been implicated in the development of sepsis and multiple organ dysfunction syndrome (MODS) increasing morbidity and mortality in burn patients [4]. In addition to LPS, most enteric Gram-negative bacteria secrete flagellin, which may also play a pathogenic role in the development of systemic inflammatory response syndrome (SIRS) and MODS following burn injury due to its translocation from the intestine into the systemic circulation.

Flagellin is the primary protein component of the flagella that is located on the outer membrane of most Gram-negative bacteria; flagellin monomers assemble into a flagellum polymer [5]. Flagellin release in the intestinal lumen is caused by a combination of 1) *de novo* synthesis and deliberate ejection of the flagellum and 2) shearing of flagella from the bacterial surface caused by host proteases, pH, temperature and/or bile salts. Flagellin monomers, in the absence of bacteria, bind to toll-like receptor 5 (TLR5) resulting in the induction of innate immune responses [6, 7] activating a wide range of cell types to induce the release of various inflammatory mediators [8–10].

The ability of flagellin to induce harmful or beneficial immune responses has been shown to depend on its concentration, the administration route, the responding cell type and the experimental model employed [5, 6]. Interestingly, small amounts of flagellin have been shown to be beneficial, in fact, flagellin is an effective vaccine adjuvant and is being tested clinically [11]. Additionally, Neely *et al* showed that administering a single low dose of flagellin in a murine model of burn injury and infection restored neutrophil response increasing bacterial clearance [12]. In contrast, others studies have shown that flagellin can induce pathophysiological inflammation that causes tissue damage [5, 9, 10, 13], a condition we have termed as flagellemia. Neutralization of flagellin can attenuate systemic inflammatory responses [14]. We, as well as others, have reported that flagellin is an active contributor to pathophysiological responses such as sepsis [5, 9, 10, 12, 15, 16], liver injury [13], pulmonary inflammation and cardiovascular damage [9, 16, 17, 18]. We recently reported that luminal flagellin is internalized by human IECs [6], followed by dissemination into the systemic circulation under various pathophysiological conditions including burn injury [19, 20] and inflammatory bowel disease (IBD) [21–24].

In this current study we investigated whether flagellin contributes to intestinal barrier dysfunction resulting in gut-derived bacterial translocation and systemic dissemination, that can accelerate a systemic infection to further deteriorate an impaired host immune response following burn injury. Therefore, we used a biologically relevant *in vitro* model of polarized human intestinal epithelia whereby the apical surface represents the lumen and the basolateral surface represents the lamina propria and bloodstream. To translate our *in vitro* findings to an intact biological model, we also used a well-established murine model of burn injury to examine the role of gut-derived flagellin in systemic inflammation and distal organ damage. Our experiments were designed using selected doses of flagellin in combination with the intestinal commensal *E*. *coli* (EC) strain O83:H1, which does not harbor pathogenic virulence genes [25], to determine whether flagellin functions as a primary contributor to intestinal dysfunction and systemic inflammation or is merely a consequence of excessive inflammation that typically follows burn injury.

## Methods

### Burn patient serum and flagellin ELISA

Flagellin was detected and quantified by ELISA (NovaTein Bio; Woburn, MA) in the sera from five male burn patients without a burn-related infection, ranging in age from 9–15 years old with an average total body surface area (TBSA) burn of 37.7% (Table 1). All, but one, patient was provided peri-operative vancomycin or imnepinem upon hospital admission (Table 1). Sera from four unburned, healthy individuals served as controls. The collection of the serum was approved by the University of Texas Medical Branch (UTMB) Institutional Review Board (IRB; 04–157) and was obtained via written consent (form was approved by UTMB IRB under 04–157) from volunteer burn patients at Shriners Hospitals for Children (Galveston, Texas).

**Table 1:** Flagellin detected in pediatric burn patient serum and healthy donors.

Patient	TBSA %	Burn Type	Sera collection days after burn injury	Patients receiving peri- op antibiotics	Flagellin ng/mL (Burn)	Flagellin ng/mL (Healthy)	Healthy Donor
**1**	**43**	**Electric/Flame**	**5 days**	**Yes**	**337**	**195**	**1**
**2**	**39**	**Scald/Flame**	**2 days**	**Yes**	**349**	**175**	**2**
**3**	**40**	**Electric/Flame**	**4 weeks**	**Yes**	**378**	**205**	**3**
**4**	**23**	**Flame/Inhalation**	**12 days**	**No**	**876**	**116**	**4**
**5**	**43**	**Flame/Inhalation**	**11 days**	**Yes**	**1589**	**---**	**---**
				**Average Flagellin**	*705±**181**	**173**±**14**	

* p<0.05 compared to healthy donor sera

### Bacteria and flagellin

The human intestinal bacterial isolate *E*. *coli* O83:H1 [25] was generously provided by Dr. Alexander Swidsinski (Charite Hospital, Germany). The bacteria was grown overnight in 10 mL of modified Brain Heart Infusion (BHI) broth at 37°C with agitation. The overnight culture was pelleted by centrifugation and then suspended in sterile phosphate-buffered saline (PBS) at a concentration of 1 × 10^9^ colony-forming units (CFU)/mL. The prepared stock then was diluted further in sterile PBS to the desired concentrations. Bacterial ten-fold dilutions were plated on brain heart infusion (BHI) plates to confirm experimental dosage. The bacterial dose for the *in vitro* and *in vivo* experiments herein was determined by our previous study [26]. Ultrapure *Salmonella typhimurium* flagellin was purchased from Invivogen (San Diego, CA).

### Intestinal cell line, cell stimulation, and analysis of cell supernatants

Human colon adenocarcinoma intestinal epithelial cells (Caco-2BBe; American Type Culture Collection, Rockville, MD) were grown in Dulbecco’s modified Eagle’s medium (DMEM) supplemented with 10% fetal bovine serum (FBS), 110 mg/mL sodium pyruvate, and antibiotics (100 IU of penicillin/mL and 100 ug of streptomycin/mL). For experiments, Caco-2BBe cells were grown on 3 um transwell filters (Corning, Inc.; Corning, NY). All experiments were performed using serum- and antibiotic-free culture medium to prevent interference with the flagellin protein and/or bacterial interaction with the host cell as previously described [6]. Caco-2BBe cells were stimulated apically with 50 or 500 ng of ultrapure *Salmonella typhimurium* flagellin (Invivogen) and 1 x 10^5^ EC as follows: 1) flagellin was added 30 minutes (min) prior to the addition of EC [S1 Fig], 2) flagellin and EC were added simultaneously to cells [S1 Fig], or 3) EC was added 30 min prior to flagellin [S1 Fig]. Cells and treatment alone served as controls. Apical and basolateral supernatants were collected at 6h post-stimulation and analyzed by IL-8 ELISAs (Thermo Scientific^™^ Pierce^™^; Grand Island, NY). Additionally, to determine bacterial and flagellin translocation from the apical to the basolateral chamber, dilutions of the basolateral supernatant were plated on BHI plates and incubated overnight to determine individual colony counts. Flagellin levels in the basolateral chamber were quantified by ELISA (NovaTein Bio; Woburn, MA).

### IkB-alpha degradation analyzed by western blot

Caco-2BBe cells, grown in 6-well plates, were stimulated with the same flagellin and EC treatments as stated above and analyzed via Western blot [6]. Caco-2BBe cells then were lysed in 4°C buffer containing 50 mM of Tris (pH 8.0), 110 mM of NaCl, 5 mM of EDTA, 1% Triton X-100, and 0.1 mM of PMSF. The amount of protein in each sample was determined by the Bradford assay (Bio-Rad, Hercules, CA). Individual cell lysates were heated to 100°C in 10 uL of loading buffer (4% SDS, 20% glycerol, 125 mM Tris-HCl (pH 6.8), and 10% 2-ME), and 40 ug of each protein sample was loaded per lane on an 8–16% Tris-glycine gradient gel (NOVEX, San Diego, CA). Electrophoresed proteins were transferred to a nitrocellulose membrane (NOVEX) with the NOVEX X-cell MiniGel system. Membranes were blocked with 10% nonfat dried milk suspended in TBS for 30 min at room temperature (RT) before incubation with rabbit polyclonal anti-IkB-alpha antiserum (Santa Cruz Biotechnology, Santa Cruz, CA) at a dilution of 1:1000 for 3h at RT. Blots were washed twice for 7 min in TBS supplemented with 0.1% Tween 20 + TBS, followed by the addition of peroxidase-conjugated anti-rabbit IgG (Sigma-Aldrich, St. Louis, MO) at a dilution of 1:10,000 for 1h at RT. Blots were washed three times for 5 min each with TBS plus Tween 20 and then incubated for 1 min in ECL reagents at RT (ECL kit; Amersham, Little Chalfont, Buckinghamshire, U.K.). Processed blots were placed on x-ray film (Kodak, Rochester, NY) for empirically optimized exposures.

### HEK293 cells expressing Toll-like receptors (TLRs) stimulated by *E*. *coli* and/or flagellin

As described previously [27], TLR5 expressing human embryonic kidney (HEK) 293 cells (Invivogen, San Diego, CA) were cultured and maintained in Dulbecco's modified Eagle's medium (DMEM) (Cellgro Mediatech Inc., Herndon, VA) supplemented with 10% FBS, 100 units/ml penicillin, 100 ug/ml streptomycin, and 2 mM glutamine. To analyze the activation of NF-κB, the HEK293 cells were transfected with 2.5 ug of the firefly luciferase (NF-κB) reporter plasmid pNiFty2-Luc (Invivogen) using Lipofectamine 2000 (Invitrogen, Carlsbad, CA). The transfected cells were then plated at 1 x 10^4^ cells (60% confluence) in 24-well format culture plates. The day after plating, the existing medium was replaced with 100 uL of DMEM containing no antibiotics, followed by addition of 50 or 500 ng of ultrapure flagellin and/or 1 x 10^5^ CFU of EC as described above. DMEM alone (with no cells) and untreated HEK293 cells were used as negative controls. Samples were analyzed for luciferase activity by the SuperLight luciferase reporter gene assay (BioAssay Systems, Hayward, CA) as described by the manufacturer and measured using a SpectraMax M2 spectrophotometer (Molecular Devices, Sunnyvale, CA). The data were expressed as relative luciferase activity.

### Animals and burn injury model

Eight-week old female Balb/c mice weighing between 19–21 grams (Jackson Laboratories) were housed in an Association for Assessment and Accreditation for Laboratory Animal Care (AAALAC)-approved housing facility and permitted to adjust to their environment for 7 d prior to procedures receiving free access to food and water throughout the study. All procedures were approved by the University of Texas Medical Branch Institutional Animal Care and Use Committee and performed humanely with minimal suffering. The animals (n = 5–7 mice/group) were anesthetized with a standard dose of ketamine/xylazine then gavaged with 1 ug/mouse (50 ug/kg) or 5 ug/mouse (250 ug/kg) of ultrapure *S*. *typhimurium* flagellin (Invivogen) 2h prior to burn, simultaneous with burn or 2h post-burn. Subsequently, mice were subjected to a 30% TBSA burn injury as described previously [28]. Sham burned mice (e.g. shaved, anesthetized but not burned) served as controls. Mice were euthanized at 24 or 48h post-burn and serum MLN, intestine and lungs were sterilely collected from each animal. A small section of the lower lung was collected from each animal and assessed for lung histopathology via H&E staining. MLN and lungs were homogenized and murine monocyte chemoattractant protein-1 (MCP-1) and murine chemoattractant CXCL1/KC (the functional murine equivalent of human IL-8) were quantified via ELISA (R&D Systems; Minneapolis, MN).

### Gut microbiome analysis

DNA from sterilely harvested intestinal samples (3mm^3^ pieces), collected from three animals per group as described in the previous section. Each tissue was placed directly into 350 ul of MagnaPure bacterial lysis solution (Roche) and then snap frozen on dry ice before storage at -80^°^C until DNA was extracted. Samples were thawed and then subjected to both sonication and proteinase steps to enhance complete genomic isolation prior to automated extraction in a MagnaPure96 magnetic bead system (Roche; Indianapolis, IN). Isolated DNAs were amplified with a panel of 5 “universal” fusion primer pairs that create overlapping 400-500bp DNA fragments covering 95% of the bacterial 16S gene. The bar coded (Ion Xpress Barcodes) amplimers were mixed at equal ratios and then subjected to Ion Torrent NGS using the associated reagents (ThermoFisher Scientific Inc, Waltham, MA). Average read length was >300 bases with sequence produced from both strands increasing confidence. For the vast majority of bacteria, all 5 contigs were produced by the PCR (data not shown). Subsequent database evaluations required that at least two contig regions were identified as the same bacterial genus or species to include the hit in the dataset (i.e. a minimum of ~500 bp of contiguous sequence in the 16S gene). NGS reads were filtered for quality and binned using the Ion Torrent Suite software (v 4.0.2). Sequencing reads in FASTQ format were then further processed using web-based Galaxy software where each barcoded read was trimmed to remove the primer sequence; the 16S sequences then were compared to the SILVA 16S database using bowtie 2 software to identify species or genera as well as the number of times each sequence matched the database (hit-rate). Where multiple calls to the same genera were made the number of hits were added accordingly. These numbers were then converted to percentage of total to give an overall ratio of the sequenced microbiome. We completed ~20 distinct sequence runs as foundational data to identify the most common bacterial elements in both the normal and burned murine intestinal microbiome.

## Results

### Flagellin is present in the serum of burn patients

We analyzed pediatric burn patient sera for the presence of flagellin. Our results showed that significantly higher flagellin levels were detected in the sera of burn patients compared to healthy donor sera (Table 1; p<0.05). Flagellin levels in the serum of individual burn patients varied markedly (Table 1); the highest levels of flagellin were detected in the serum of patients with flame burn injuries accompanied by inhalation injury (Table 1; Burn Patient Serum [BPS] 2 and 3) compared to electrical (Table 1; BPS 1 and 4) or scald burns (Table 1; BPS 5). The average level of flagellin in healthy controls was 173 ± 14 compared to 705 ± 181 pg/ml in burn patients. It is noteworthy, that the second highest level of flagellin was detected in the serum of a burn patient with the smallest TBSA (23%) who received no peri-operative antibiotics (Table 1). These data show elevated, but variable levels of flagellin in burn patient serum regardless of the type of burn injury or the day of serum collection post-burn supporting our basic supposition that circulating flagellin, or flagellemia, may associate with disease states in burn patients.

### The timing and dose of flagellin significantly impacted basolateral cytokine secretion and gut-derived bacterial and flagellin translocation

The presence of flagellin in the serum of burn patients led us to investigate the role of flagellin from luminal or systemic sites upon innate immune responses following burn injury. Under pathophysiological conditions in the intestine following burn injury (e.g. oxygen deprivation to tissue and organ, ischemia/reperfusion), the apical surface of IECs are exposed to higher levels of gut-derived flagellin and luminal bacteria. To determine if flagellin is a primary contributor to intestinal dysfunction or merely a consequence of inflammation following burn injury we applied flagellin alone or in combination with EC to the apical surface of polarized Caco-2 cells. Our initial studies evaluated a low dose of flagellin (50 ng) tested with and without 1 x 10^5^ cfu of EC as follows: 1) flagellin was added 30 min prior to EC, 2) flagellin and EC (flagellin/EC) were added simultaneously or 3) EC was added 30 min prior to flagellin (see S1 Fig). Untreated cells served as controls. Basolateral supernatants were collected 6h after treatments and analyzed for IL-8 secretion and translocation of EC and flagellin from the apical to the basolateral chamber.

**Fig 1:**
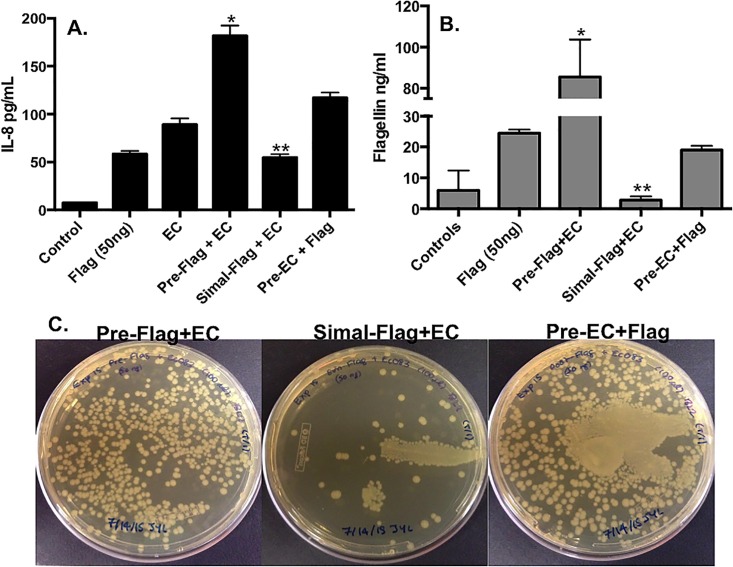
Flagellin (50 ng) and *E*. *coli* induced time-dependent secretion of IL-8 that correlated with translocation. **(A)** Pre-Flag+EC induced significantly higher levels of IL-8 vs. all other groups (*p<0.05) while Simal–Flag+EC induced significantly less IL-8 secretion vs. Pre-Flag+EC and Pre-EC+Flag (**p<0.05). **(B)** Flagellin translocated from the apical to basolateral surface at a significantly higher rate in the Pre-Flag+EC group vs. all other groups (*p<0.05) and flagellin translocation occurred significantly less in the Simal–Flag+EC vs. all other groups except controls (**p<0.05). Data is represented as mean±SEM * denotes significance of (p<0.05). **(C)** The translocation of EC occurred higher in the Pre-Flag+EC and Pre-EC+Flag vs. Simal–Flag+EC.

The results showed that apical EC induced generally higher levels of IL-8, compared to 50 ng of flagellin (Fig 1A). Interestingly, IL-8 levels were dependent on the order of exposure. Specifically, flagellin added to IECs prior to EC synergistically induced maximal levels of basolateral IL-8 secretion, compared to all other groups (Fig 1A; p<0.05). Conversely, EC added prior to flagellin induced only moderate levels of IL-8 compared to flagellin added prior to EC, while simultaneous addition of flagellin/EC to IECs stimulated significantly lower levels of IL-8 (Fig 1A; p<0.05).

As an indicator of barrier integrity, we evaluated translocation of both flagellin and EC in polarized IEC cultures. The data indicated that IL-8 secretion correlated with the levels of translocated flagellin and EC detected in the basolateral chamber. The highest level of flagellin (Fig 1B; p<0.05 vs. all other groups) and EC (Fig 1C) translocation occurred when flagellin was added prior to EC. Modest flagellin translocation was detected after IEC were treated with flagellin alone suggesting some impact on barrier integrity. Similar levels of basal flagellin were observed in cultures where EC was added prior to flagellin (Fig 1B). Finally, simultaneous application of flagellin and EC induced the lowest translocation of flagellin (Fig 1B; p<0.05) and EC (Fig 1C). Together the data support the conclusion that IL-8 secretion by IECs correlated to translocation levels of both bacterial and flagellin and was dependent on the order of apical application supporting the underlying hypothesis that flagellin can have inflammatory or anti-inflammatory effects that correlate with barrier integrity.

**Fig 2:**
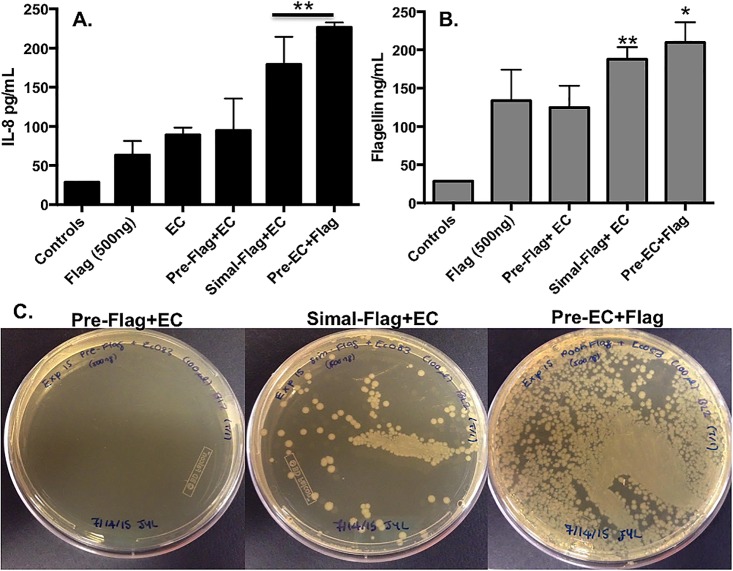
Flagellin (500 ng) and *E*. *coli* synergistically induced time-dependent secretion of IL-8 and translocation. **(A)** The Simal–Flag+EC and Pre-EC+Flag groups induced significantly higher levels of IL-8 vs. all other groups (**p<0.05). **(B)** Flagellin translocated from the apical to basolateral surface was maximal in the Pre-EC+Flag group vs. all other groups (*p<0.05) while flagellin translocation occurred at a significantly higher rate in the Simal–Flag+EC group vs. all other groups (**p<0.05) except Pre-EC+Flag. Data is represented as mean±SEM. **(C)** The translocation of EC occurred at the highest level in the Pre-EC+Flag group with modest EC translocation Simal–Flag+EC group vs. little to no translocation detected in the Pre-Flag+EC group.

Next, we evaluated the intraluminal effects of a 10x higher dose of flagellin (500 ng) using the same paradigm described above. The data for IL-8 elaboration showed distinct patterns from the lower dose evaluations. Specifically, high dose flagellin added prior to EC showed less synergism stimulating IL-8 levels that were only marginally higher than flagellin alone and were equal to EC alone (Fig 2A). Maximal IL-8 secretion was observed when EC was applied prior to flagellin with significant IL-8 secretion induced by simultaneous application of high dose flagellin and EC (Fig 2A; p<0.05). Elevated flagellin (Fig 2B; p<0.05) and EC (Fig 2C) translocation again correlated with increased IL-8 secretion in cells simultaneously stimulated with flagellin and EC or when EC were added prior to flagellin.

These data suggest that the direct contact of the luminal intestinal surface with a normal enteric EC strain prior to or accompanied by 500 ng of flagellin collaborate to induce elevated levels of IL-8 and the translocation of both EC and flagellin. The lower dose studies confirmed that these effects were dependent upon the flagellin levels at the luminal IEC surface. The intensity of intestinal inflammation and translocation also was dependent on the application of independent components alone or in combination (flagellin *vs*. EC *vs*. both) and the timing of their administration.

### Flagellin in combination with a normal gut flora EC isolate induced different patterns of IkB-alpha degradation and NF-κB activation in IECs in a time and dose dependent manner

**Fig 3:**
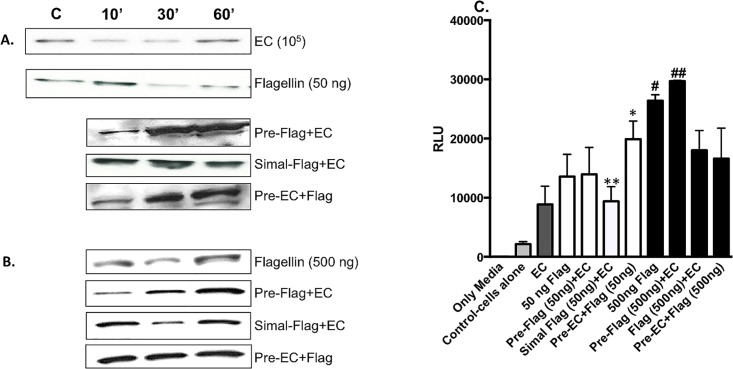
Flagellin and *E*. *coli* O83:H1 induced differential degradation of IkB-alpha and NF-κB activation via TLR5. Western blot analysis detected IkB-alpha degradation in IECs as follows: **(A)** IECs stimulated with EC alone, 50 ng of flagellin or various combination of both stimuli. **(B)** IECs were stimulated with the same stimulus regime as in **(A)** except using 500 ng of flagellin. **(C)** The results showed that NF-κB activation was modest in cells stimulated with EC while NF-κB activation was higher in cells stimulated with 50 ng of flagellin alone compared to EC alone. Pre-EC+Flag [50ng] functioned synergistically to induce significant NF-κB activation compared to Simal-EC+flagellin [50ng], EC or flagellin [50ng] alone (*p<0.05). NF-κB activation was significantly lower in the Simal-EC+flagellin [50ng] compared to 50 ng of flagellin alone, Pre-Flag [50ng]+EC and Pre-EC+Flag [50ng] (**p<0.05). A 10-fold increase in its dose to 500 ng induced significant NF-κB activation compared to 1 x 10^5^ of EC (# p<0.05). NF-κB activation was maximal in cells stimulated with 500 ng of flagellin prior to EC (Pre-Flag [500ng]+EC) compared to Simal+Flag [500 ng]+EC or Pre-EC+Flag [500 ng] (## p<0.05).

Flagellin monomers released from Gram-negative bacteria bind to TLR5 that signals NF-κB activation through IkB-alpha degradation resulting in the production of various inflammatory mediators including IL-8 [5, 6, 29, 30]. To examine the mechanism of differential secretion of IL-8 and subsequent barrier dysfunction following exposure to gut-derived flagellin and EC, we examined IkB-alpha/NF-κB signaling pathway in IECs treated as described above. Apical application of EC (10^5^) alone induced degradation of IkB-alpha at 10 and 30 min post-stimulation with IkB-alpha replenishment at 60 min (Fig 3A). Conversely, 50 ng of flagellin alone stimulated IkB-alpha degradation with prolonged degradation extending beyond 60 min (Fig 3A). The combination of flagellin and EC at various times induced differential signal transduction via IκBα. Specifically, following the addition of 50 ng of flagellin prior to EC or vice versa induced marked IκBαdegradation at 10 min with robust renewal of IkB-alpha at 30 and 60 min (Fig 3A). Alternatively, little to no IkB-alpha degradation was seen following simultaneous flagellin and EC application at any of the tested time points (Fig 3A) suggesting a modulatory effect on cell signaling whereby the phosphorylation and degradation of IkB-alpha is diminished subsequently leading to muted IL-8 secretion (Fig 1A).

However, as shown in Fig 3B, a higher dose of flagellin (500 ng) induced IkB-alpha degradation at 30 min with IkB-alpha replenishment at 60 min. Flagellin added prior to or simultaneously with EC induced IkB-alpha degradation at 10 and 30 min, respectively, with renewal by 60 min (Fig 3B). Interestingly, EC added prior to flagellin failed to induce IkB-alpha degradation at any of the tested time points (Fig 3B) which does not correlate with IL-8 secretion observed in Fig 2A.

To investigate the stimulation of alternative receptor(s) and the induction of signal transduction pathway(s) by EC, we confirmed TLR5 activation utilizing human TLR5-expressing HEK-293 cells that were transfected with an NF-κB/luciferase reporter plasmid. Cells were stimulated with flagellin and EC as described above. At 6h post-stimulation, NF-κB activation was measured as luciferase activity. The results showed that NF-κB activation was modest in cells stimulated with EC (10^5^) which suggests that EC is stimulating alternative receptor(s). EC added prior to flagellin functioned synergistically to induce significant NF-κB activation compared to simultaneous EC/flagellin, EC or flagellin alone (Fig 3C; p<0.05). Further, NF-κB activation was significantly lower in cells stimulated with simultaneous EC/flagellin compared to 50 ng of flagellin alone, pre-flagellin/EC or pre-EC/flagellin (Fig 3C; p<0.05).

In contrast to 50 ng of flagellin, a 10-fold increase in its dose to 500 ng induced significant NF-κB activation compared to 1 x 10^5^ CFU of EC (Fig 3C; p<0.05). Furthermore, NF-κB activation was maximal in cells stimulated with 500 ng of flagellin prior to EC compared to simultaneous flagellin/EC or EC added prior to flagellin (Fig 3C; p<0.05). Interestingly, the presence of EC simultaneous with or prior to flagellin diminished NF-κB activation (Fig 3C) while IL-8 secretion increased (Fig 2A and 2B).

These data demonstrated that EC influenced flagellin’s induction of IkB-alpha degradation and subsequent NF-κB activation. This suggests that EC is stimulating receptor(s) other than TLR5 and stimulating (an) alternative pathway(s) to contribute to cytokine secretion [31–35].

### Gut-derived flagellin induced systemic inflammation and produced gut microbiome shifts following burn injury

**Fig 4:**
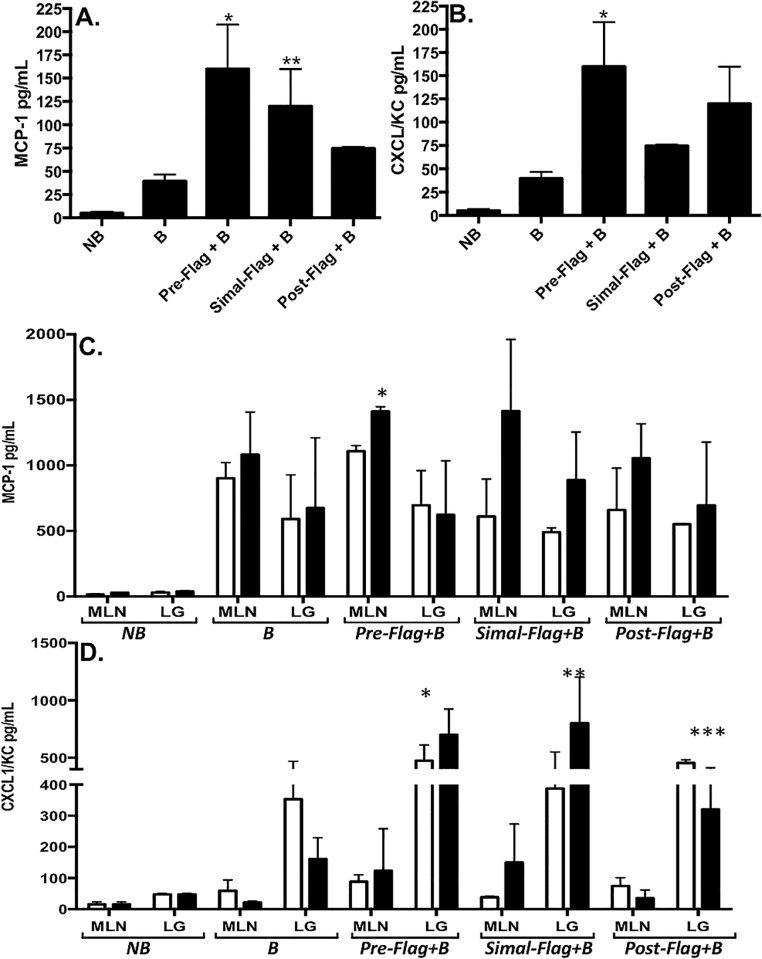
Intraluminal flagellin induced systemic cytokine secretion following burn injury. To characterize systemic inflammation, levels of MCP-1 (**A** and **C**) and CXCL1/KC (**B** and **D**) were quantified by ELISA from serum collected 48h post burn injury (**A** and **B**) or MLN and lung tissues (**C** and **D**) collected at 24 (white bars) or 48h (black bars) after burn. Groups are as described in the methods and results including sham, non-burned controls (*NB*), 30% TBSA injury (*B*), flagellin delivered by gavage 2h prior to burn (*Pre-Flag+B*), simultaneously to burn (*Simal-Flag+B*) or 2h after burn (*Post-Flag+B*). Data are represented as mean±SEM from at least 5 animals in a group. Comparisons with statistically different values are denoted as follows: (**A**) MCP-1 levels were significantly higher in the Pre-Flag+B group vs. sham burn, burn alone and Post-Flag+B (*p<0.05) while Simal-Flag+B induced significantly higher MCP-1 levels vs. sham burn and burn alone (**p<0.05). (**B**) CXCL1/KC was significantly higher in the Pre-Flag+B group compared to sham burn, burn alone and Simal-Flag+B (*p<0.05). (**C**) Pre-Flag+B induced significantly higher levels of MCP-1 in the MLNs vs. burn alone (*p<0.05). CXCL1/KC was significantly higher in the lungs of the Pre-Flag+B at 24 and 48h compared to burn alone (*p<0.05). (**D**) At 24h, CXCL1/KC was significantly higher in the lungs of the Post-Flag group compared to burn alone (***p<0.05) while the Simal-Flag+B group had markedly higher levels of CXCL1/KC in their lungs at 48h compared to burn alone (**p<0.05).

As shown in Table 1, burn patient sera illustrated flagellemia relative to healthy donors. Based on these results we investigated whether systemic, translocated gut-derived flagellin may lead to intestinal microbiome shifts associated with systemic inflammation following burn injury. For these studies we again employed our burned mouse model where controlled introduction of flagellin (1 ug dosing) via gavage could be more effectively studied. Systemic inflammation was characterized by quantification of cytokine levels in sera and relevant tissues from mice that were euthanized 24 or 48h post-burn. Sham, non-burned (NB) and 30% TBSA alone (B) mice served as controls (Fig 4). We focused our evaluations on two proteins, MCP-1 and IL-8, previously described as biomarkers for inflammation related to burn injury in pediatric patients [54]. Several distinct comparisons are detailed below in effort to highlight specific interpretations from these complex datasets associated with the progressive responses seen in our mouse model.

As expected, at both the 24 and 48h time points, burn or flagellin alone produced significant elevations in both MCP-1 (Fig 4A and 4C) and CXCL1/KC (Fig 4B and 4D) in sera (panels A and B) and lung and MLN tissues (panels C and D) relative to the samples from non-burned control animals subjected to the sham-burn procedure (NB; p<0.05). Comparing tissues levels at both time points, MLN samples generally showed higher levels of MCP-1 than corresponding lung samples and, conversely, lung tissues had higher levels of CXCL1/KC to MLNs (Fig 4). Further, the MLN response generally peaked at 24h while the highest levels in the lung were consistently detected in the 48h post burn samples.

Together, these results illustrated the progression of the effects created by each of the conditions where burn alone showed distinct outcomes to the addition of flagellin before or after burn. Specifically, MCP-1 was significantly elevated in the serum of the 2h pre-flagellin/burn compared to burn and 2h post-flagellin/burn group averages (Fig 4A; p<0.05). Mice gavaged with flagellin simultaneously to the burn injury or 2h after burn both produced significantly higher levels of MCP-1 in the serum compared to burn mice alone (p<0.05). At both 24 and 48h time points, significantly higher levels of MCP-1 were detected in the MLN of the 2h pre-flagellin/burn compared to burn injury alone (Fig 4C; p<0.05). Flagellin delivered simultaneously to or 2h after burn led to lower levels of MCP-1 in the MLNs collected at 24h compared to burn alone suggesting an initial tempering of MCP-1 production. This effect was not evident in the MLNs from the same groups at 48h. MCP-1 levels in the lungs of each of the flagellin groups were not significantly different from burn alone.

CXCL1/KC, the murine IL8 equivalent, was elevated to significantly higher levels in serum samples from mice gavaged with flagellin 2h prior to burn relative to burn injury alone (Fig 4B; p<0.05). Simultaneous gavage of flagellin with burn injury produced significantly lower levels than flagellin delivered 2h before the burn but the detected levels were still significantly higher than burn alone (Fig 4B; p<0.05). Flagellin 2h after burn produced serum levels of CXCL1/KC that were intermediate to the other flagellin deliveries. In MLN tissues, no obvious increases were detected relative to burn alone animals at both time points. In lung samples, mice that received flagellin showed higher levels of CXCL1/KC relative to burn alone (Fig 4D; p<0.05). The animals gavaged with flagellin 2h prior to burn had the highest levels of this cytokine in the lungs of any group. Most interestingly, CXCL1/KC showed an increase in levels at 48h for both the 2h prior and the simultaneous gavage of flagellin while burn alone or flagellin 2h post burn led to a reduction in CXCL1/KC at 48h. These trends were not significant but notable in terms of understanding the impact of flagellin’s presence relative to burn.

**Fig 5:**
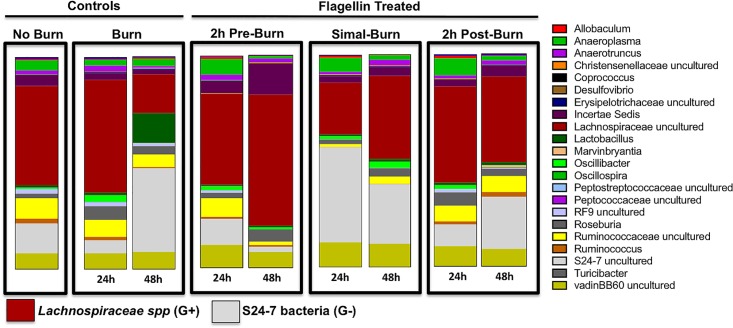
Intraluminal flagellin induced changes in the gut microbiome following burn injury. The most marked changes in the gut microbiome following burn injury were in the families of Gram-positive *Lachnospiraceae spp* and Gram-negative S24-7 bacteria. Burn alone showed minimal changes to gut microbiome profiles. At 24h, however, there was a marked increase in S24-7 and decrease in *Lachnospiraceae spp* at 48h post-burn compared to sham burn. Flagellin gavaged 2h prior to burn (Pre-flagellin) induced a substantial increase in *Lachnospiraceae spp* and a decrease in S24-7 bacteria at 48h post-burn compared to sham controls. Fagellin just prior to burn injury stimulated S24-7 abundance with a decrease in *Lachnospiraceae spp* at 24h post-burn compared to non-burned controls. Finally, flagellin gavaged 2h post-burn (Post-flagellin) induced minimal changes in the gut microbiome at 24h but at 48h *Lachnospiraceae spp* decreased with a coincident increase in S24-7 bacteria.

The collective results from the murine studies suggested that both inflammation and barrier integrity changes likely would shift the intestinal microbiome where additional bacteria and their products could translocate to exacerbate burn complications. Using a novel next generation sequencing approach that captured sequence for over 90% of the bacterial 16S genes of community members present, we observed that burn injury and/or flagellin administration induced microbial community changes relative to the sham injury control mice (Fig 5). The most dramatic change in the gut microbiome following burn injury were in the abundant families of the S24-7 group of Gram-negative Bacteroidales and Gram-positive Lachnospiraceae family bacteria. We observed only minor changes in these gut bacterial families in burned mice at 24h following burn, however, at 48h post-burn an increase in the abundance of S24-7 bacteria and decreases in Lachnospiraceae were observed compared to sham mice (Fig 5). Conversely, at 48h there was a substantial increase in the abundance of *Lachnospiraceae spp* with a parallel decrease in S24-7 group members in mice gavaged 2h pre-burn (Fig 5). When gavaged immediately prior to burn, flagellin induced an increase in the S24-7 proportion and a decrease in *Lachnospiraceae spp* at 24h post-burn whereas this trend was reversed by 48h post-burn (Fig 5). Finally, the relative abundance of the *Lachnospiraceae spp* in mice gavaged with flagellin 2h post-burn moderately decreased at 48h compared to 24h post-burn/flagellin and sham burn controls (Fig 5).

Collectively our data indicate that, during burn injury, gut-derived flagellin is a primary instigator of systemic inflammation and contributes to gut microbiome changes that may further impact intestinal barrier integrity.

### Gut-derived flagellin and EC synergistically induce systemic inflammation

**Fig 6:**
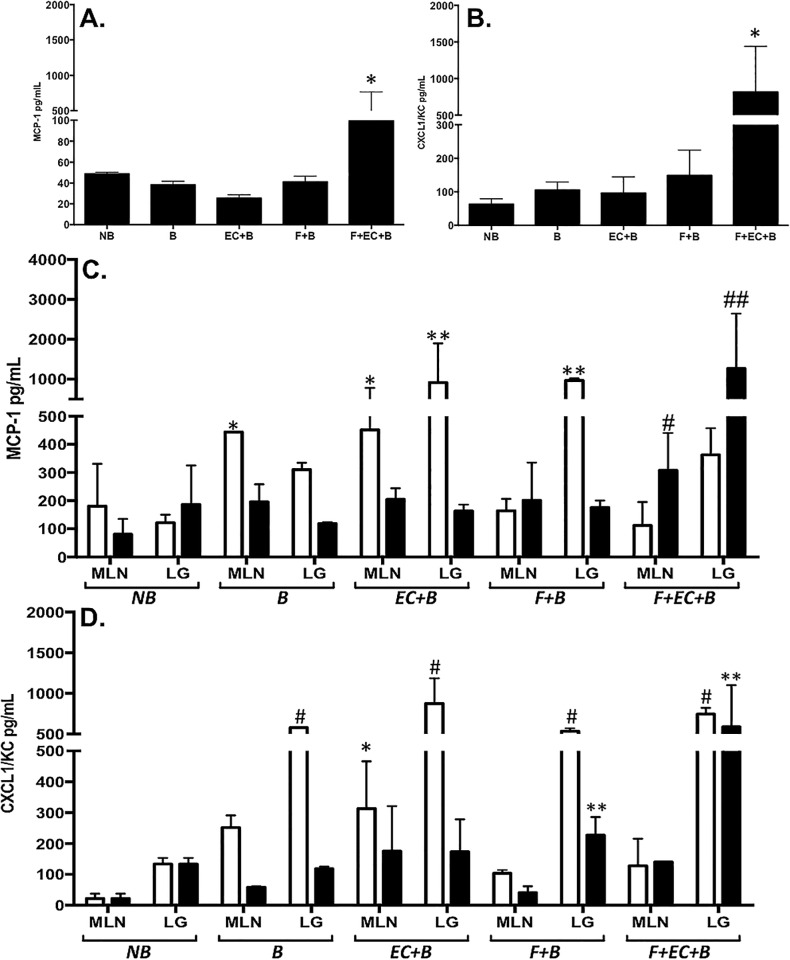
Intraluminal flagellin and *E*. *coli* O83:H1 synergistically induced systemic cytokine production following burn injury. Mice were gavaged with 5 ug of flagellin and/or 1 X 10^5^ CFU EC 2h prior to burn. Animals were humanely euthanized at 24 (white bars) or 48h (black bars) post-burn and serum (**A** and **B**), MLNs and lungs (**C** and **D**) were collected and analyzed for MCP-1 (**A** and **C**) and CXCL1/KC (**B** and **D**) by ELSIA. Groups are as described in the methods and results including sham, non-burned controls (*NB*), 30% TBSA injury (*B*), EC and 30% TBSA (*B+EC*), 5ug flagellin and 30% TBSA (*B+F*) and the gavaged combination of EC and flagellin prior to burn (*B+F+EC*). Data are represented as mean±SEM from at least 5 animals in a group. Comparisons with statistically different values are denoted as follows: (**A & B**) MCP-1 and CXCL1/KC were significantly higher in the serum of the *B+EC+F* group vs. all other groups (*p<0.01). (**C**) MCP-1 was significantly higher in the MLNs of *B* and *B+EC* at 24h compared to *NB* (*p<0.05). MCP-1 was significantly elevated in the lungs of *B+EC* and *B+F* groups at 24h compared to *B* lungs (**p<0.05). *B+EC+F* induced maximal levels of MCP-1 in the MLNs and lungs at 48h vs. all other groups (#p<0.05). (**D**) CXCL1/KC was significantly higher in the lungs of all groups compared to non-burned (#p<0.05). CXCL1/KC was significantly higher in the MLNs of *B+EC* mice compared to all other groups (*p<0.01). *B+F* and *B+EC+F* groups had significantly higher levels of CXCL1/KC in the lungs at 48h vs. all other groups (**p<0.05).

To assess the effects of flagellin in combination with the normal gut isolate, EC O83:H1 (EC) on inflammation, mice were gavaged with flagellin (5 ug dosing) and/or 1 x 10^5^ EC cfu/mouse then subjected to a 30% TSBA injury 2h post-gavage. Sham burn and burn alone mice again served as controls. Mice were euthanized at 24 and 48h post-burn to provide sera (48h only) and MLN and lung tissues at 24 or 48h after burn injury. Levels of MCP-1 (Fig 6A and 6C) and CXCL1/KC (B and D) were quantified showing that the flagellin and EC prior to the burn injury gavage led to the seen in any of our studies (p<0.01 compared to all other groups; Fig 6A and 6B) supporting the conclusion that the combination of a normal gut bacteria with a high dose of flagellin produced extreme systemic inflammation.

Distinct from our studies with 1ug gavage of flagellin (Fig 4), the higher dose of flagellin in these studies as well as the addition of 10^5^ CFU of EC had more pronounced effects on the lung than the MLN with regard to expression levels of both MCP-1 and CXCL1/KC (Fig 6C and 6D). Further, maximal levels of MCP-1 were detected at 48h in the MLNs and lungs (Fig 6C; p<0.01) of the EC/flagellin/burn group compared to all other groups suggesting a prolonged or increasing inflammatory process in this group (Fig 6C; p<0.05). For the other groups, MCP-1 generally declined at 48h in both tissues. Finally, the higher dose of flagellin did lead to a higher level of MCP-1 in the lungs at 24h (Fig 6C) than the lower dose tested (Fig 4C); the remaining time points were quite similar for the two flagellin doses.

**Fig 7:**
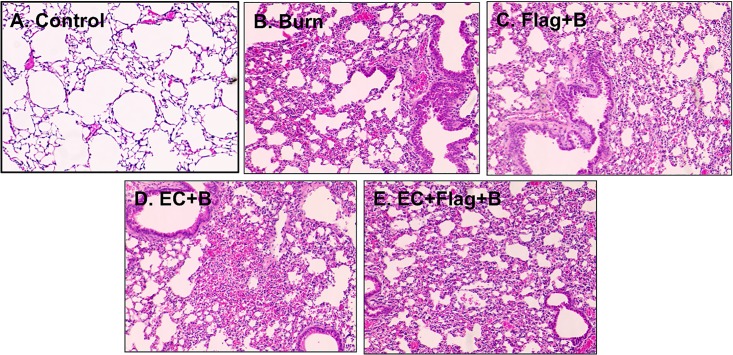
Intraluminal flagellin and *E*. *coli* O83:H1 synergistically induced lung damage following burn injury. Lungs were collected at 48h post-burn from mice as described in Fig 6. Compared to normal, healthy controls (**A**) significant cell infiltration and disruption of the alveolar structure was observed in mice receiving burn, flagellin and burn, EC and burn, or all three insults (**B-D**). All three insults also induced intra-alveolar hemorrhaging and prominent cell accumulation indicated lung parenchymal injury (**E**). Mag 20X.

The levels of CXCL1/KC expression in all the burned and gavaged animals were significantly higher at 24h compared to the non-burned group (Fig 6D; p<0.05). The CXCL1/KC expression patterns from the animals that were gavaged with both flagellin and EC prior to burn containing significantly higher levels of this cytokine at 48h in the lung tissues than other groups (Fig 6D; p<0.05). The higher dose of flagellin produced similar outcomes to the lower dose in both tissues (Figs 4D and 6D) for CXCL1/KC. The histological examination of lungs from the same mice receiving burn (B; Fig 7B), flagellin (500 ng) and burn (Flag; Fig 7C), EC and burn (Fig 7D), or a combination of all three (Fig 7E) showed disruption of the alveolar architecture with the accumulation of inflammatory cells, and alveolar wall injury compared to normal, healthy controls (Fig 7A). While lungs from mice receiving burn, flagellin and EC (Fig 7E) also showed signs of intra-alveolar hemorrhage and fluid-filled alveoli likely a result of inflammatory cell infiltration (Fig 7E). These results correlate with systemic inflammation induced by flagellin and EC following burn injury. Collectively, these data indicated that flagellin and EC in the intestinal luminal function synergistically to induce systemic inflammation, that is more pronounced in the lungs, following burn injury.

## Discussion

Our research contributes to the understanding of the involvement of gut-derived flagellin in systemic post-burn pathogenesis *in vitro* and *in vivo*. In this study, we demonstrated that flagellin levels were increased in the sera of pediatric burn patients compared to healthy donors. Additionally, our *in vitro* and *in vivo* findings showed that intraluminal flagellin was a determinant of gut dysbiosis and systemic inflammation following burn injury. The data revealed that intraluminal flagellin induced systemic inflammation predominantly in the lungs. These data suggest that flagellin can cause primary as well as secondary damage to an already immune-compromised burn patient. The therapeutic relevance of our current results have the potential of establishing a novel therapeutic paradigm whereby preventing the binding of flagellin to TLR5, predominately expressed in the lungs and liver, may reduce morbidity and mortality in pediatric burn patients.

Because flagellin is commonly released/shed into the surrounding environment by flagellated Gram-negative bacteria, including commensal flora, there is an opportunity for the protein to come into direct contact with the apical surface of intestinal epithelial cells. We have previously shown that in small amounts, luminal flagellin aids in the maintenance of gut homoeostasis and can serve as a potential “warning” signal to the host by inducing moderate, controlled innate immune responses via TLR5 that are beneficial and protective to the host [6, 11, 12]. However, under pathophysiological conditions, monomeric flagellin can stimulate pathogenic inflammation causing tissue and organ damage [5, 9, 10, 13, 18].

The lack of blood flow to the intestine following burn injury can allow excessive amounts of commensal gut flora and their products, including flagellin, access to the apical surface of IECs leading to escape of the intestinal contents [6, 28] and damaging systemic inflammation [9, 10, 15, 36, 37]. As such, we detected varying but significantly higher levels of flagellin in the sera of pediatric burn patients compared to healthy donors. Flagellin levels were likely affected by the type and severity of burn injury but our cohort was too small to support any specific associations. The age of the patient and the day the serum was collected post-burn likely also contribute to variations in flagellin levels that could be addressed in future, larger cohort studies. The detected flagellin could not be confirmed as intestinal in origin, but did provide the necessary clinical association between burn injury and flagellemia necessary to support the examination of subsequent systemic inflammation in burn patients. We were unable to analyze the burn patient sera for cytokine secretion to correlate with flagellin levels due to small sample volumes. However, in future studies we will obtain and analyze larger volume sera samples in triplicate to compare the level of flagellin and cytokine secretion.

To explore the impact of flagellemia and commensal bacteria we extended our investigations to our controlled IEC *in vitro* and mouse model,that revealed post-burn consequences of both intra-luminal and systemic nature. The culmination of our findings showed that the exposure of intestinal epithelial cells to luminal bacteria and flagellin induced inflammation in a dose and time-dependent manner. Specifically, exposure of IECs to a small amount of flagellin prior to and in combination with EC decreased the secretion of basolateral IL-8, which preserved the gut barrier as demonstrated by decreased EC and flagellin translocation out of the intestine. These results correlate with a recent report demonstrating that intraperitoneal administration of 0.125 ng of flagellin, prior to burn injury and infection restores the neutrophil population that led to increased clearance of *P*. *aeruginosa* after wound inoculation [12]. Further, another report suggested that, before an insult on the host, the presence of flagellin at low levels affords protection [38] conceivably by moderating innate immunity in order to prepare the host for an impending assault [6]. We have extended these reports showing that higher levels of luminal flagellin combined with EC to synergistically provoke barrier dysfunction and excessive inflammation.

It has been shown previously that commensal flora are capable of stimulating as well as interfering with TLR signaling pathways thereby altering pro-inflammatory responses [31–35, 39, 40]. Although TLR stimulation is under tight control to avoid harmful inflammation in response to normal flora, commensals do express factors that can trigger innate immune responses via TLRs and other receptor signaling pathways [39]. However, following burn injury, damage to the intestinal tract can lead to the loss of commensal tolerance and subsequently allow IECs to be overexposed to normal flora and their products resulting in gut dysbiosis [40, 41]. Our data showed that EC moderately stimulated TLR5 and diminished responses to flagellin. As such, enteric EC may stimulate alternative receptor-signaling pathway(s) to induce IL-8 secretion in IECs. While future experiments are needed to identify this pathway, Zargar *et al* [42] reported that two genetically similar strains of nonpathogenic *E*. *coli* strains stimulated IL-8 and TNF secretion from IECs while also upregulating negative-feedback regulators in NF-κB and NOD-like signaling pathways. Likewise, Gram-negative gut commensals have been reported to circumvent TLR stimulation and instead activate NF-κB via the Nod1-signaling pathway [34, 35].

Although it has been reported that low doses of flagellin prior to an insult (e.g. radiation treatment, bacterial infections and chemical exposure) to the host stimulated protective innate immune responses [38], high levels of systemic flagellin triggered pathophysiological responses in specific inflammatory diseases [13]. Therefore, in the second part of our study, we utilized a well-established mouse model of thermal injury to delineate the effects of intraluminal flagellin on the intestinal microbiome and it’s subsequent escape from the damaged intestine following burn injury. We observed that intraluminal flagellin caused a shift in the gut bacterial communities depending on the time flagellin was present following burn injury. The most marked changes were seen when flagellin was present several hours prior to or simultaneous with burn injury. Interestingly, the intraluminal presence of flagellin prior to burn injury promoted the overgrowth of the Gram-positive *Lachnospiraceae spp* accompanied by a decrease in the Gram-negative S24-7 bacteria. It has been suggested that *Lachnospiraceae spp* are potentially protective bacteria that may be linked to the maintenance of gut barrier function [43]. Moreover, a reduction in Lachnospiraceae bacteria has been reported in IBD patients [44]. Recently, Earley *et al* [41] reported an abundance of *Enterobacteriaceae spp* in the intestine of burned mice that translocated to the MLN. In these same mice, *Lachnospiraceae spp* were decreased. In the current study, we did not find that *Lachnospiraceae spp* abundance preserved gut barrier function, as the presence of flagellin in the lumen prior to burn injury caused an increase in systemic inflammation in the serum, MLN and lungs. However, flagellin given simultaneous to burn injury induced a shift in the mouse microbiome to the Gram-negative S24-7 bacterial group with decreased *Lachnospiraceae spp* subsequently followed by systemic inflammation.

Although sequencing technology has advanced the ability to link various pathophysiological conditions with distinct intestinal microbial profiles, it is still problematic to identify the “good” and “bad” normal gut flora. Various reports suggest that Lachnospiraceae is a “good” family of bacteria, others have shown that the over-abundance of Lachnospiraceae in females was associated with earlier onset and/or more severe lupus symptoms [45]. Moreover, the gut microbial profile of patients with an inflammatory disease of joints, ankylosing spondylitis, had significantly greater abundance of *Lachnospiraceae spp* and increased intestinal permeability compared to healthy individuals [46, 47]. Likewise, it was recently reported that the interaction of enteric commensal viruses and bacteriophages with normal flora contributes to gut bacterial-viral immune tolerance/homeostasis [48, 49]. While we did not investigate the role of enteric viruses in this study, current reports [48, 49] as well as the changes we observed in the gut microbiome following flagellin gavage and burn injury, support the hypothesis that enteric viruses may contribute to gut dysbiosis and/or systemic inflammation. Additional causative studies will be required to conclusively delineate what combination of enteric bacterial species or viruses are responsible for gut homeostasis or to blame for disease development. Further, such studies also could address the possibility that overgrowth of a normal flora that is considered probiotic could promote gut dysbiosis [48, 49, 50]. Our current data support the conclusion that intraluminal flagellin alters the gut microbiome in a mouse burned model, that produced gut dysbiosis [50], flagellin translocation and systemic inflammation.

The groundbreaking work by Deitch *et al* [51, 52] on gut dysfunction following trauma showed that gut-derived factors carried in the intestinal MLN contribute to distant organ injury. Injuries in the range of 30–40% TBSA, can causes excessive cytokine secretion with the most significant increases in IL-6, IL-8, MCP-1, MIP-1β, and G-CSF that can be a measure of patient outcome [53, 54]. The lung is the first organ exposed to the intestinal contents contained in the MLN, thus it is the first organ to fail in severely injured patients [51–53]. The mechanism of lung injury includes injurious systemic inflammation (e.g. activated neutrophils and endothelial permeability/cell death) initiated by gut-derived factors present in MLN tissues including flagellin [51]. High levels of flagellin, ranging from 10 to 200 ug, in the circulation are dangerous to the host due to TLR5 expression differences [13]; the lungs are particularly vulnerable to flagellin exposure due to high expression levels of TLR5 [2, 4, 51–53]. Together these findings may explain the observed impact of a modest amount of intraluminal flagellin (1 or 5 ug/mouse) on MCP-1 and CXCL1/KC production in the lungs. Our subsequent findings that the combination of flagellin and EC exacerbated inflammation in and damage of the lungs suggests that this synergism may be the more important contributor to lung injury and systemic inflammation post-burn. Importantly, the data indicate that burn patients are more susceptible to damage from small amounts of gut-derived bacteria and their products.

In conclusion, the present study demonstrated that gut-derived flagellin alone or accompanied by an intestinal commensal EC strain acts as an initiator of luminal and systemic inflammation following burn injury. Both the intraluminal and systemic pathological effects of flagellin were dependent on the dose and order flagellin and EC were recognized by IECs. Our data highlight the complex pathophysiological role of gut-derived flagellin in burn patients. These findings may stimulate further work in the area to develop therapeutic interventions using a single, appropriately selected anti-flagellin antibody (i.e. one that targets its conserved regions to prevent binding of flagellin to TLR5) that would function synergistically with other successful burn treatments to stabilize the intestinal barrier and modulate MODS and SIRS.

## Supporting Information

S1 Fig Scenarios for human intestinal epithelial cell contact with flagellin and *E*. *coli* O83:H1. Caco-2BBe cells were grown on transwell filters to a confluent, polarized monolayer then exposed to flagellin and *E*. *coli* O83:H1 as follows: **A)** flagellin contacting the apical surface of cells prior to *E*. *coli*, **B)** flagellin and *E*. *coli* contacting cells simultaneously, or **C)** *E*. *coli* contacting cells prior to flagellin.
(TIF)

## Abbreviations

EC: *E*. *coli* O83:H1
Flag: Flagellin
IEC: Intestinal epithelial cells
LG: lungs
MLN: Mesenteric lymph nodes
Simal: Simultaneous
TBSA: total burn surface area
TLR5: Toll-like receptor 5
