# Correction: Intraluminal Flagellin Differentially Contributes to Gut Dysbiosis and Systemic Inflammation following Burn Injury

**DOI:** 10.1371/journal.pone.0170722

**Published:** 2017-01-19

**Authors:** Logan Grimes, Allie Doyle, Aaron L. Miller, Richard B. Pyles, Gabor Olah, Csaba Szabo, Sarah Hoskinson, Tonyia Eaves-Pyles

The seventh author’s name is spelled incorrectly. The correct name is: Sarah Hoskinson.
