# Supplementary figures and images for: Intraluminal Flagellin Differentially Contributes to Gut Dysbiosis and Systemic Inflammation following Burn Injury
Source: PLoS One. 2016 Dec 1;11(12):e0166770. doi: 10.1371/journal.pone.0166770 (PMC5131931; doi:10.1371/journal.pone.0166770)

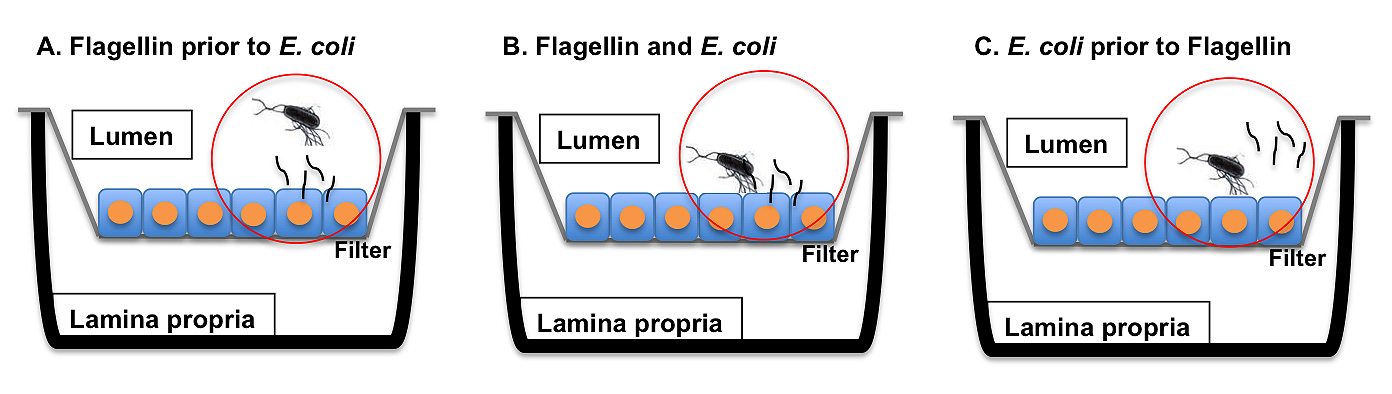

Supplement: S1 Fig — Caco-2BBe cells were grown on transwell filters to a confluent, polarized monolayer then exposed to flagellin and E. coli O83:H1 as follows: A) flagellin contacting the apical surface of cells prior to E. coli, B) flagellin and E. coli contacting cells simultaneously, or C) E. coli contacting cells prior to flagellin. (TIF) [file pone.0166770.s001.tif]
